# Comparative Remission Rates and Tolerability of Drugs for Generalised Anxiety Disorder: A Systematic Review and Network Meta-analysis of Double-Blind Randomized Controlled Trials

**DOI:** 10.3389/fphar.2020.580858

**Published:** 2020-11-11

**Authors:** Wenqiang Kong, Huiyuan Deng, Jie Wan, Yilu Zhou, Yan Zhou, Bihui Song, Xiuling Wang

**Affiliations:** ^1^ Department of Pharmacy, Zi Gong First People’s Hospital, Zi Gong, China; ^2^ Department of Pharmacy, Children’s Hospital of Chongqing Medical University, Chongqing, China; ^3^ National Clinical Research Center for Child Health and Disorders, Children’s Hospital of Chongqing Medical University, Chongqing, China; ^4^ Ministry of Education Key Laboratory of Child Development and Disorders, Children’s Hospital of Chongqing Medical University, Chongqing, China; ^5^ China International Science and Technology Cooperation Base of Child Development and Critical Disorders, Children’s Hospital of Chongqing Medical University, Chongqing, China; ^6^ Chongqing Key Laboratory of Pediatrics, Children’s Hospital of Chongqing Medical University, Chongqing, China

**Keywords:** remission rate, tolerability, pharmacotherapy, network meta-analysis, generalized anxiety disorder

## Abstract

**Background**: Generalized anxiety disorder (GAD) is one of the most common psychiatric disorders associated with substantial dysfunction and socioeconomic burden. Pharmacotherapy is the first choice for GAD. Remission [Hamilton Anxiety Scale (HAM-A) score ≤7] is regarded as a crucial treatment goal for patients with GAD. There is no up-to-date evidence to compare remission rate and tolerability of all available drugs by using network meta-analysis. Therefore, the goal of our study is to update evidence and determine the best advantageous drugs for GAD in remission rate and tolerability profiles.

**Method**: We performed a systematic review and network meta-analysis of double-blind randomized controlled trials (RCTs). We searched PubMed, EMBASE, Cochrane Central Register of Controlled Trials, Chinese National Knowledge Infrastructure, wanfang data, China Biology Medicine and ClinicalTrials.gov from their inception to March 2020 to identify eligible double-blind, RCTs reporting the outcome of remission in adult patients who received any pharmacological treatment for GAD. Two reviewers independently assessed quality of included studies utilizing the Cochrane Collaboration’s risk of bias tool as described in Cochrane Collaboration Handbook and extracted data from all manuscripts. Our outcomes were remission rate (proportion of participants with a final score of seven or less on HAM-A) and tolerability (treatments discontinuations due to adverse events). We calculated summary odds ratios (ORs) and 95% confidence intervals (CIs) of each outcome via pairwise and network meta-analysis with random effects.

**Results**: Overall, 30 studies were included, comprising 32 double-blind RCTs, involving 13,338 participants diagnosed as GAD by DSM-IV criteria. Twenty-eight trials were rated as moderate risk of bias, four trials as low. For remission rate, agomelatine (OR 2.70, 95% CI 1.74–4.19), duloxetine (OR 1.88, 95% CI 1.47–2.40), escitalopram (OR 2.03, 95% CI 1.48–2.78), paroxetine (OR 1.74, 95% CI 1.25–2.42), quetiapine (OR 1.88, 95% CI 1.39–2.55), and venlafaxine (OR 2.28, 95% CI 1.69–3.07) were superior to placebo. For tolerability, sertraline, agomelatine, vortioxetine, and pregabalin were found to be comparable to placebo. However, the others were worse than placebo in terms of tolerability, with ORs ranging between 1.86 (95% CI 1.25–2.75) for tiagabine and 5.98 (95% CI 2.41–14.87) for lorazepam. In head-to-head comparisons, agomelatine, duloxetine, escitalopram, quetiapine, and venlafaxine were more efficacious than tiagabine in terms of remission rate, ORs from 1.66 (95% CI 1.04–2.65) for duloxetine to 2.38 (95% CI 1.32–4.31) for agomelatine. We also found that agomelatine (OR 2.08, 95% CI 1.15–3.75) and venlafaxine (OR 1.76, 95% CI 1.08–2.86) were superior to vortioxetine. Lorazepam and quetiapine were poorly tolerated when compared with other drugs.

**Conclusions**: Of these interventions, only agomelatine manifested better remission with relatively good tolerability but these results were limited by small sample sizes. Duloxetine, escitalopram, venlafaxine, paroxetine, and quetiapine showed better remission but were poorly tolerated.

## Introduction

Generalized anxiety disorder (GAD) is a typically chronic mental disorder characterized by excessive, uncontrollable, and persistent worrying and tension. It is associated with clinical manifestations including palpitations, tremor, restlessness, fatigue, and difficulty concentrating, among others, which cause marked functional impairment across multiple aspects of productivity, family activity and socialization and associated reduced quality of life (Doyle and Pollack, 2003; Tyrer and Baldwin, 2006; Baldwin D. S. et al., 2011). In Europe the 12-months prevalence of GAD is approximately 0.2–4.2% and the life prevalence is approximately 4.3–5.9% (Wittchen and Jacobi, 2005). In urban China, the prevalence of GAD also has been estimated to be approximately 2.4–8.9%. Among those patients, one third self-reported receiving no therapy or even counsels (Yu et al., 2018). Therapies include pharmacological treatment, psychological treatment, or a combination of both.

Psychological interventions for GAD such as cognitive behavior therapy are widely considered preferable to anxiolytic drugs because of their efficacy and harmlessness, but often they cannot be implemented due to limited resources (Gould et al., 1997; Tyrer et al., 2006). Pharmacological therapy is probably still the main clinical treatment for GAD. In clinical practice, clinicians may have difficulty prescribing optimal drugs and facing various obstacles. In a previous survey, Yu and colleagues reported that diazepam, pregabalin, and alprazolam were the most common prescription medications for GAD in urban China, although selective serotonin reuptake inhibitors (SSRIs) and serotonin and norepinephrine reuptake inhibitors (SNRIs) have been indicated as first-line treatments for GAD in guidelines and by meta-analyses (Yu et al., 2018; National Institute For Health and Care Excellence, 2011; Slee et al., 2019).

Achieving response is the traditional goal of GAD therapy and such responses has been defined as either a clinically significant improvement or a meaningful reduction in HAM-A scale or Clinical Global Impressions (CGI) scale score, but many patients exhibit residual symptoms and are at a high risk of recurrence after initially responding to therapy (Mandos et al., 2009; Baldwin D. S. et al., 2011). Thus, the ultimate treatment goal is complete remission with no symptoms of anxiety in addition to complete recovery to premorbid functioning (Doyle and Pollack, 2003; Mandos et al., 2009). In a mixed-treatment meta-analysis comparing nine drugs in 27 trials published between 1980 and 2009 fluoxetine exhibited the best remission rate, and sertraline exhibited the highest tolerability (Baldwin D. et al., 2011). Since that meta-analysis, several new antidepressants such as agomelatine, vilazodone and vortioxetine have demonstrated considerable effects on anxiety symptoms. Therefore, the current network meta-analysis was conducted to compare all available drugs in patients with GAD using data from double-blind, randomized, controlled trials.

## Materials and Methods

Our systematic review and network meta-analysis were performed in accordance to the checklist of the Preferred Reporting Items for Systematic Reviews and Meta-analyses (PRISMA) extension statement for reporting of systematic reviews incorporating network meta-analyses of healthcare interventions (Hutton et al., 2015).

### Search Strategy and Study Selection

Seven electronic databases including PubMed, EMBASE, the Cochrane Central Register of Controlled Trials, the Chinese National Knowledge Infrastructure, wanfang data, China Biology Medicine, and ClinicalTrials.gov were systematically searched from their inception to March 2020 to identify trial reports. The search terms used were “anxiety”, “anxiety disorder”, “generalized anxiety disorder”, “randomized controlled trials”, and “RCT”. The references lists of relevant meta-analyses, reviews, pooled analyses, and included trials were also reviewed to obtain additional studies. The languages were limited to Chinese and English. Unpublished trials were excluded, because the reliability of data derived from them could not be assured. The search strategy is presented in detail in Table 1.

**TABLE 1:** Search strategies.

Electronic databases	Search strategies
Pubmed	#17 #5 and #16
	#16 14 NOT #15
	#15 (“Animals” [Mesh]) NOT “Humans” [Mesh]
	#14 #6∼13 or
	#13 groups [tiab]
	#12 trial [tiab]
	#11 randomly [tiab]
	#10 drug therapy [sh]
	#9 placebo [tiab]
	#8 randomized [tiab]
	#7 controlled clinical trial [PT]
	#6 randomized controlled trial [PT]
	#5 #1∼4 or
	#4 generali* anxiety disorder
	#3 GAD
	#2 anxiety disorder
	#1 “Anxiety Disorders” [Mesh]
CENTRAL	#5 #1∼4 or
	#4 generali* anxiety disorder
	#3 GAD
	#2 anxiety disorder
	#1 anxiety disorder [MeSH]
Embase	#10 #5 and #9
	#9 #6∼#8 or
	#8 double-blind:ti,ab
	#7 placebo:ab,ti,lnk
	#6 random:ti,ab
	#5 #1∼4 or
	#4 generali* anxiety disorder
	#3 GAD
	#2 anxiety disorder
	#1 “anxiety disorder”/exp
ClinicalTrials.gov	Anxiety disorder

^*^represents truncation searching.

Two reviewers (KWQ, WXL) independently conducted study selection in accordance with pre-specified inclusion criteria, and any disagreements was settled via discussion. After removing duplicates, they then screened the titles and abstracts of remaining records, read the remaining reports in full text and identified eligible studies.

### Inclusion Criteria

The inclusion criteria were 1) double-blind randomized controlled trials (RCTs) comparing active drugs with placebo or another agent as oral monotherapy in adults with a primary diagnosis of GAD with major comorbidities except those with major depression disorder, substance abuse, schizophrenia, organ diseases or alcohol addiction; 2) standard diagnostic criteria included Diagnostic and Statistical Manual of Mental Disorders, Third Edition (DSM-III), Diagnostic and Statistical Manual of Mental Disorders, Fourth Edition (DSM-IV), Diagnostic and Statistical Manual of Mental Disorders, Fifth Edition (DSM-V), International Classification of Diseases, 9th Revision (ICD-9), International Classification of Diseases, 10th Revision (ICD-10) or Chinese Classification of Mental Disorders, Third Edition (CCMD-3); 3) remission rates were reported. Articles reporting studies investigating refractory GAD, relapses or changing to another drug were excluded.

### Data Extraction

Two investigators (KWQ, WXL) independently extracted data using pre-designed data extraction forms. The data extracted from each report included basic study characteristics (first author, publication year, study duration, total sample size, attrition rate, sponsor), baseline patient characteristics (sex ratio, mean HAM-A, mean age, diagnostic criteria), interventions (drugs and doses), and outcomes (remission rate and tolerability). Remission rate was defined as the proportion of patients who had achieved remission (HAM-A scores ≤7) at the study end-point. Tolerability was determined based on treatment discontinuations due to adverse events. The intention-to-treat (ITT) population consisting of all randomized participants who received at least one dose of study medication was abstracted for two outcomes. In cases of missing or unclear data, first authors or corresponding authors were contacted via email for supplementary information. Any discrepancies were settled by discussion.

### Risk of Bias Assessment

The quality of the trials included was assessed in accordance with the Cochrane Collaboration’s risk of bias tool as described in the Cochrane Collaboration Handbook (Higgins et al., 2011). Two investigators (KWQ, WXL) independently determined risks bias to be low, unclear, or high based on the presence or absence of random sequence generation (selection bias), allocation concealment (selection bias), blinding of participants and personnel (performance bias), blinding of outcome assessors (detection bias), incomplete outcome data (attrition bias), selective reporting (reporting bias), and “other source of bias” (other bias). Subsequently, we divided study quality into three rates from low risk to high risk on the basis of the method described by two articles (Cipriani et al., 2018; He et al., 2019). Discrepancies were resolved via discussion.

### Data Analysis

Pairwise meta-analyses using RevMan5.2 software were performed first, to compare the results of mixed treatment meta-analyses. Summary odds ratios (ORs) with 95% confidence intervals (CIs) were calculated via Mantel-Haenszel’s method. A random-effects model was used to derive pooled estimates across studies, because it takes between-study differences into account. Between-study heterogeneity was quantitatively assessed using the *I* ^2^ statistic, with *I* ^2^ of >50% indicating high heterogeneity and <50% indicating low heterogeneity (Higgins and Thompson, 2002).

Network meta-analyses were then performed using STATA software. A frequentist framework was applied to combine evidence from direct and indirect comparisons with a random effects model (Chaimani et al., 2013). Loop inconsistency was assessed in every closed triangular or quadratic loop via the “loop-specific” approach, wherein a 95% CI excluding zero suggests that the loop is inconsistent (Higgins et al., 2012; Li et al., 2017). The “design-by-treatment” interaction model was used to assess global consistency in networks (Li et al., 2017). The surface under the cumulative ranking curve (SUCRA) and the mean ranks were calculated to rank the treatments for each outcome (Salanti et al., 2011). The comparison-adjusted funnel plots were generated to investigate whether there are study-small effects in the intervention network (Chaimani and Salanti, 2012). The robustness of conclusions was evaluated via Bayesian analysis.

## Results

### Study Selection and Study Characteristics

**FIGURE 1:**
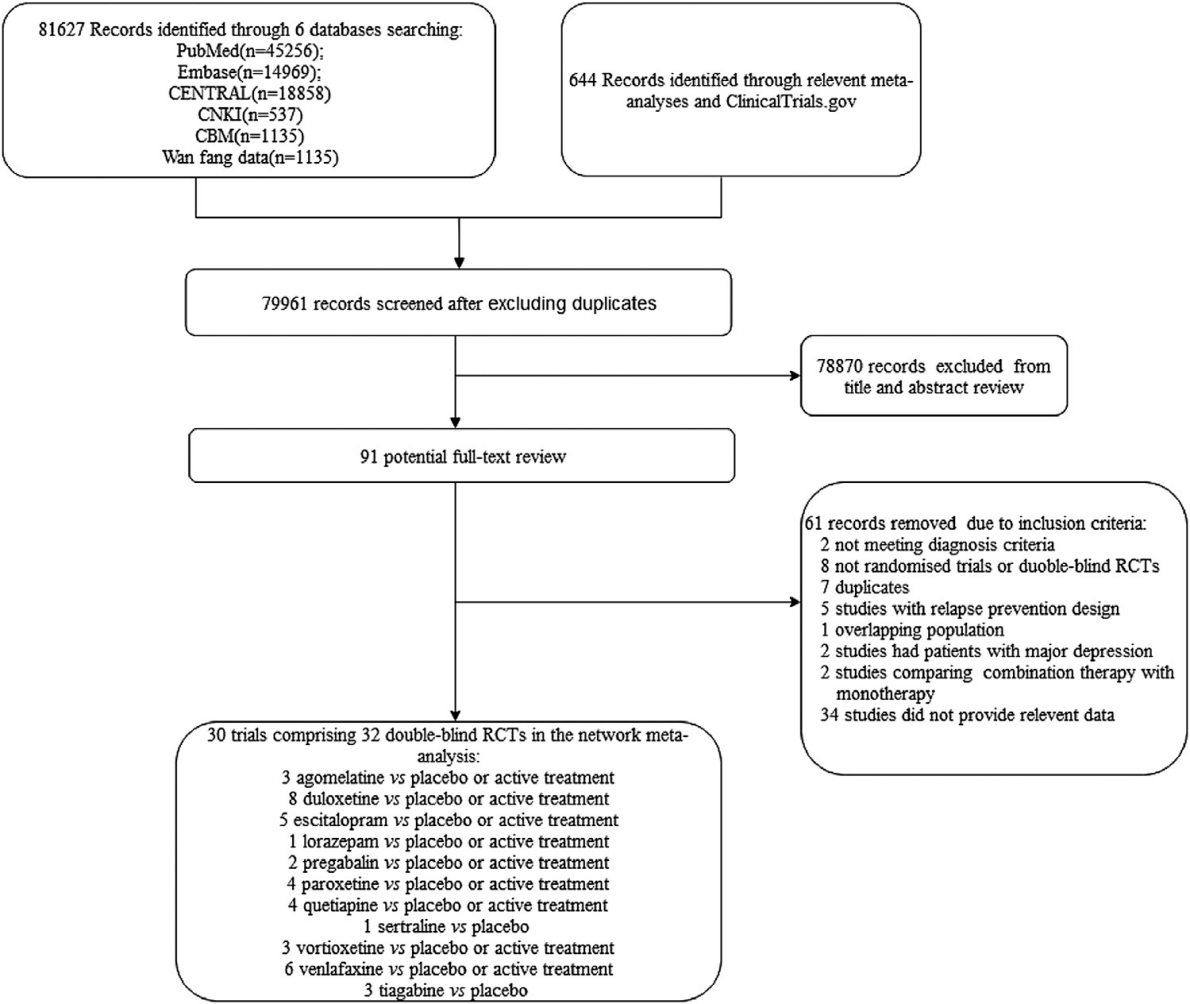
Flow diagram of study selection.

The literature screening process is shown in Figure 1. Electronic searches yielded 82,271 citations, and the full text versions of 91 publications were subsequently reviewed. Of these, 61 were rejected based on the inclusion criteria. Thirty studies (Pollack et al., 2001; Feltner et al., 2003; Lenox-Smith and Reynolds, 2003; Rickels et al., 2003; Allgulander et al., 2004; Boyer et al., 2004; Davidson et al., 2004; Nimatoudis et al., 2004; Hartford et al., 2007; Koponen et al., 2007; Bose et al., 2008; Montgomery et al., 2008; Pollack et al., 2008a; Pollack et al., 2008b; Rynn et al., 2008; Stein et al., 2008; Nicolini et al., 2009; Bandelow et al., 2010; Coric et al., 2010; Khan et al., 2011; Wu et al., 2011; Bidzan et al., 2012; Merideth et al., 2013; Mezhebovsky et al., 2012; Rothschild et al., 2012; Alaka et al., 2014; Kasper et al., 2014; Mahableshwarkar et al., 2014; Stein et al., 2014; Ball et al., 2015; Stein et al., 2017) comprising 32 double-blind RCTs were ultimately included in the network meta-analysis and all of them were published in English.

Basic information derived from each of the 32 RCTs is shown in Table 2. Collectively they involved 13,338 participants diagnosed via the DSM-IV, published between 2003 and 2017. Of these participants 4,848 were randomly assigned to a placebo group and 8,490 were randomly assigned to an active medication group. Thirteen drugs or placebo were included in the analysis. The study’s sample sizes ranged from 46 to 951.60.0% (8,007/13,338) of the participants were female. The vast majority of patients were Caucasian. The medication dosage was flexible in 17 trials. The majority of participants had moderate-to-severe GAD, with a mean HAM-A scale score between 22.6 and 29.0. The adult patient groups had mean ages between 36.3 and 72.4 years. Dropout rates ranged from 6.9 to 50%. Four studies exclusively included people aged >65 years (Montgomery et al., 2008; Mezhebovsky et al., 2013; Alaka et al., 2014; Ball et al., 2015). The study durations ranged from 4 to 24 weeks (median 10 weeks). All studies were placebo-controlled trials. Fourteen of the thirty-two trials (43.8%) randomly allocated patients to three or more groups, and 28/32 trials (87.5%) were funded by pharmaceutical companies.

**TABLE 2:** Characteristic of included studies.

References	Sample size	Interventions	Main race	Female (%)	Mean HAMA	Mean age (Year)	Attrition rate (%)	Sponsor	Follow-up time (weeks)	Diagnosis criteria
Lenox-Smith et al., 2003	244	Venlafaxine:75 ∼ 150 mg/day	—	61.5	28.0	48.0	13.2	Wyeth	24	DSM-IV
		Placebo		56.6	28.0	46.0	19.8			
Mahableshwarkar et al., 2014	781	Vortioxetine:2.5 mg/day	White	69.9	25.3	39.2	23.1	Takeda	8	DSM-IV
		Vortioxetine:5 mg/day		64.1	25.0	37.7	25.0			
		Vortioxetine:10 mg/day		67.3	25.3	39.8	28.8			
		Duloxetine:60 mg/day		72.4	25.0	39.5	32.1			
		Placebo		65.0	24.4	36.8	22.9			
Khan et al., 2011	951	Quetiapine:50 mg/day	White	57.1	24.6	39.0	30.8	AstraZeneca	8	DSM-IV
		Quetiapine:150 mg/day		62.8	24.5	40.7	36.1			
		Quetiapine:300 mg/day		60.7	24.5	41.0	42.3			
		Placebo		65.8	24.9	39.2	29.8			
Feltner et al., 2003	271	Pregabalin:600 mg/day	White	50.0	25.4	36.3	30.3	Pfizer	4	DSM-IV
		Lorazepam:6 mg/day		58.8	24.7	39.2	47.1			
		Placebo		50.7	24.8	37.8	28.4			
Stein et al., 2014	412	Agomelatine:25–50 mg/day	—	74.8	28.6	43.6	16.5	Servier	12	DSM-IV
		Escitalopram:10–20 mg/day		68.3	28.6	41.2	26.7			
		Placebo		71.8	28.2	43.0	17.6			
Hartford et al., 2007	487	Duloxetine:60–120 mg/day	Caucasian	64.2	25.6	40.4	45.7	Eli Lilly	10	DSM-IV
		Venlafaxine:75–225 mg/day		62.2	24.9	40.1	37.8			
		Placebo		61.5	25.0	41.9	38.5			
Stein et al., 2017	412	Agomelatine:10 mg/day	—	67.9	28.6	43.6	13.7	Servier	12	DSM-IV
		Agomelatine:25 mg/day		71.9	29.0	44.1	9.4			
		Placebo		63.4	28.8	44.1	21.1			
Alaka et al., 2014	291	Duloxetine:60–120 mg/day	Caucasian	75.5	24.6	71.4	25.0	Eli Lilly	10	DSM-IV
		Placebo		80.0	24.4	71.7	24.0			
Montgomery et al., 2008	273	Pregabalin:50–600 mg/day	White	79.0	27.0	72.4	24.9	Pfizer	8	DSM-IV
		Placebo		75.0	26.0	72.2	28.0			
Merideth et al., 2012	854	Quetiapine:150 mg/day	White	68.0	25.0	38.2	28.8	Pfizer	10	DSM-IV
		Quetiapine:300 mg/day		71.0	25.2	39.0	39.1			
		Escitalopram:10 mg/day		66.0	24.6	40.4	27.7			
		Placebo		64.0	25.3	36.6	21.4			
Stein et al., 2008	121	Agomelatine25–50 mg/day	—	68.8	29.0	42.7	8.1	Servier	12	DSM-IV
		Placebo		68.8	28.6	41.7	6.9			
Allgulander et al., 2004	378	Sertraline:50–150 mg/day	White	59.0	24.6	40.3	20.0	NA	12	DSM-IV
		Placebo		51.0	25.0	42.2	27.0			
Davidson et al., 2004	315	Escitalopram:10–20 mg/day	Caucasian	52.5	23.6	39.5	25.0	Forest laboratories	8	DSM-IV
		Placebo		52.9	23.2	39.5	22.0			
Nicolini et al., 2009	581	Duloxetine:20 mg/day	Caucasian	57.1	27.4	42.8	25.0	Eli Lilly	10	DSM-IV
		Duloxetine:60–120 mg/day					29.1			
		Venlafaxine:75–225 mg/day					27.2			
		Placebo					40			
Rickels et al., 2003	566	Paroxetine:20 mg/day	White	54.0	24.1	40.2	23.9	GlaxoSmithKline	8	DSM-IV
		Paroxetine:40 mg/day		56.0	23.8	40.5	27.4			
		Placebo		56.0	24.4	40.8	22.2			
Bandelow et al., 2010	873	Quetiapine:50 mg/day	White	68.0	26.9	40.7	25.8	AstraZeneca	8	DSM-IV
		Quetiapine:150 mg/day		66.7	26.6	42.3	25.2			
		Paroxetine:20 mg/day		64.5	27.1	41.6	20.3			
		Placebo		62.2	27.3	41.2	18.9			
Bose et al., 2008	404	Escitalopram 10–20 mg/day	White	64.6	24.2	38.2	19.7	Forest laboratories	8	DSM-IV
		Venlafaxine:75∼225 mg/day		59.7	23.8	37.1	25.6			
		Placebo		62.5	23.7	37.6	23.5			
Nimatoudis et al., 2004	46	Venlafaxine:75 mg/day	—	66.7	27.1	41.0	21	NA	8	DSM-IV
		Placebo		68.2	28.5	44.0	50			
Boyer et al., 2004	541	Venlafaxine:37.5 mg/day	—	42.0	26.6	45.0	27.1	Wyeth-Ayerst	24	DSM-IV
		Venlafaxine:75 mg/day		39.0	26.3	44.0	24.6			
		Venlafaxine:150 mg/day		35.0	26.3	45.0	22.6			
		Placebo		42.0	26.7	46.0	34.6			
Ball et al., 2015	291	Duloxetine:30–120 mg/day	Caucasian	77.7	24.5	71.6	NA	Eli Lilly	10	DSM-IV
		Placebo								
Bidzan et al., 2012	301	Vortioxetine:5 mg/day	white	68.7	26.3	45.0	14.7	Takeda	8	DSM-IV
		Placebo		61.6	26.8	45.3	16.6			
Mezhebovsky et al., 2013	450	Quetiapine 50–300 mg/day	white	72.1	25.2	70.3	20.2	AstraZeneca	9	DSM-IV
		Placebo		69.0	25.1	70.6	26.0			
Koponen et al., 2007	513	Duloxetine:60 mg/day	Caucasian	64.3	25.0	43.1	NA	Eli Lilly	9	DSM-IV
		Duloxetine:120 mg/day		72.3	25.2	44.1				
		Placebo		66.9	25.8	44.1				
Kasper et al., 2014	273	Paroxetine:20 mg/day	Caucasian	77.3	25.8	45.8	21.2	Eli Lilly	10	DSM-IV
		Placebo		73.7	25.1	44.6	13.2			
Rothschild et al., 2012	304	Vortioxetine:5 mg/day	Caucasian	67.8	24.7	41.0	17.8	Takeda	8	DSM-IV
		Placebo		63.8	24.6	41.4	25.0			
Wu et al., 2011	210	Duloxetine:60–120 mg/day	Chinese	46.3	24.5	37.3	24.1	Eli Lilly	15	DSM-IV
		Placebo		54.9	24.2	38.0	27.5			
Coric et al., 2010	157	Escitalopram 20 mg/day	White	100.0	23.5	38.8	20.8	Bristol-Myers	8	DSM-IV
		Placebo		100.0	24.7	39.3	23.1			
Rynn et al., 2008	327	Duloxetine:60–120 mg/day	Caucasian	61.3	22.6	42.2	44.6	Eli Lilly	10	DSM-IV
		Placebo		62.3	23.5	41.0	31.4			
Pollack et al., 2001	326	Paroxetine:20–50 mg/day	White	60.9	24.2	39.7	21.1	GlaxoSmithKline	8	DSM-IV
		Placebo		66.3	24.1	41.3	18.4			
Pollack et al., 2008a ^†^	910	Tiagabine:4 mg/day	—	62.0	27.0	37.6	36.0	GlaxoSmithKline	10	DSM-IV
		Tiagabine:8 mg/day		64.0	26.8	39.4	44.0			
		Tiagabine:12 mg/day		67.0	27.0	38.4	47.0			
		Placebo		67.0	26.5	38.2	37.0			
Pollack et al., 2008b ^†^	468	Tiagabine:4–16 mg/day	—	67.0	26.8	37.8	41.0	GlaxoSmithKline	10	DSM-IV
		Placebo		61.0	26.6	39.9	30.0			
Pollack et al., 2008b ^†^	452	Tiagabine:4–16 mg/day	—	61.0	27.3	39.4	29.0	GlaxoSmithKline	10	DSM-IV
		Placebo		58.0	26.7	40.8	24.0			

^†^Pollack., et al 2008 that contained three trials was considered separately.

### Risk of Bias

Random sequence generation was appropriate in 13 trials (40.6%), and allocation concealment was adequately conducted in 10 trials (31.3%). Fourteen trials (43.8%) clearly reported how they had performed the blinding of participants and personnel, and only 2 (6.3%) reported a masked outcome assessor, although all studies were double-blind RCTs. Twenty-three trials (71.9%) were considered to entail a high risk of bias based on incomplete outcome data either because they used the last observation carried forward method to handle missing data (Stack et al., 2013; Cipriani et al., 2018), or they had a high dropout rate (>20%). The risk of other bias was unclear in 28 trials (87.5%) because they were funded by pharmaceutical companies. Overall, four trials (12.5%) were rated as low risk (Stein et al., 2008; Bidzan et al., 2012; Alaka et al., 2014; Stein et al., 2014), and the other 28 were rated as moderate risk. The results of quality assessment are shown in Table 3.

**TABLE 3:** The quality assessment for included studies.

ID	Adequate sequence generation	Allocation concealment	Blinding of participant and personnel	Blinding of outcome assessment	Incomplete outcome data	Selective reporting	Other bias	Overall quality
Lenox-Smith et al., 2003	Low	Unclear	Unclear	Unclear	High	Low	Unclear	Medium^a^
Mahableshwarkar et al., 2014	Low	Low	Low	Unclear	High	Low	Unclear	Medium
Khan et al., 2011	Low	Unclear	Unclear	Unclear	High	Low	Unclear	Medium
Feltner et al., 2003	Unclear	Unclear	Low	Unclear	High	Low	Unclear	Medium
Stein et al., 2014	Low	Low	Low	Low	Low	Low	Unclear	Low^b^
Hartford., et al., 2007	Unclear	Unclear	Unclear	Unclear	High	Low	Unclear	Medium
Stein., et al., 2017	Low	Unclear	Low	Unclear	High	Low	Unclear	Medium
Alaka., et al., 2014	Low	Low	Low	Low	Unclear	Low	Unclear	Low
Montgomery., et al., 2008	Unclear	Unclear	Unclear	Unclear	Low	Low	Unclear	Medium
Merideth., et al., 2012	Unclear	Unclear	Low	Unclear	High	Low	Unclear	Medium
Stein., et al., 2008	Low	Low	Low	Unclear	Unclear	Low	Unclear	Low
Allgulander., et al., 2004	Unclear	Unclear	Unclear	Unclear	Unclear	Low	Low	Medium
Davidson., et al., 2004	Unclear	Unclear	Unclear	Unclear	High	Unclear	Unclear	Medium
Nicolini., et al., 2009	Low	Low	Unclear	Unclear	High	Unclear	Low	Medium
Rickels., et al., 2003	Unclear	Unclear	Unclear	Unclear	High	Low	Unclear	Medium
Bandelow., et al., 2010	Low	Low	Low	Unclear	High	Low	Unclear	Medium
Bose., et al., 2008	Unclear	Unclear	Unclear	Unclear	High	Low	Unclear	Medium
Nimatoudis., et al., 2004	Unclear	Unclear	Low	Unclear	High	Low	Low	Medium
Boyer., et al., 2004	Low	Unclear	Unclear	Unclear	High	Low	Unclear	Medium
Ball., et al., 2015	Unclear	Unclear	Unclear	Unclear	Unclear	Low	Unclear	Medium
Bidzan., et al., 2012	Unclear	Low	Low	Unclear	Low	Low	Unclear	Low
Mezhebovsky., et al., 2012	Low	Unclear	Low	Unclear	High	Low	Unclear	Medium
Koponen., et al., 2007	Unclear	Unclear	Unclear	Unclear	Unclear	Low	Unclear	Medium
Kasper., et al., 2014	Low	Low	Low	Unclear	High	Low	Unclear	Medium
Rothschild., et al., 2012	Unclear	Low	Low	Unclear	High	Low	Unclear	Medium
Wu., et al., 2011	Unclear	Unclear	Unclear	Unclear	High	Low	Unclear	Medium
Coric., et al., 2010	Unclear	Low	Low	Unclear	High	Low	Unclear	Medium
Rynn., et al., 2008	Unclear	Unclear	Unclear	Unclear	High	Low	Unclear	Medium
Pollack., et al., 2001	Unclear	Unclear	Unclear	Unclear	Unclear	Low	Unclear	Medium
Pollack., et al., 2008a; Pollack., et al., 2008b	Low	Unclear	Unclear	Unclear	High	Low	Unclear	Medium

a Medium-risk studies had one high-risk item or more than four items of unclear risk.

b Low-risk studies had no high-risk items and fewer than three items of unclear risk.

### Network Meta-analysis

**FIGURE 2:**
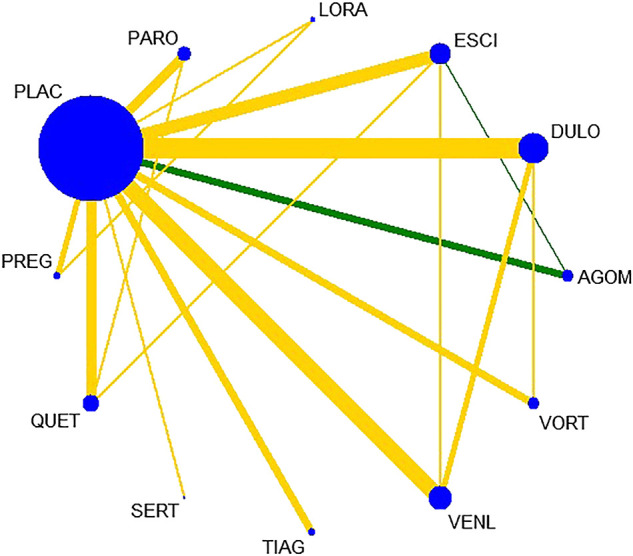
Network plot for remission rate.

**FIGURE 3:**
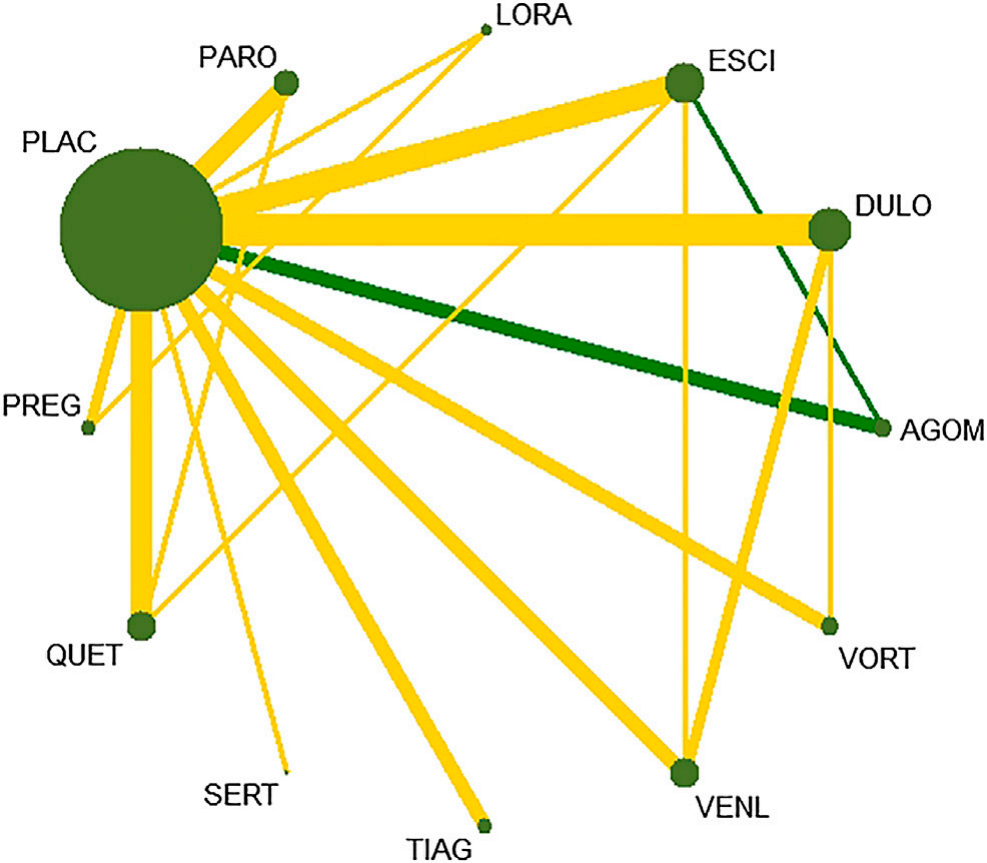
etwork plot for tolerability.

**FIGURE 4:**
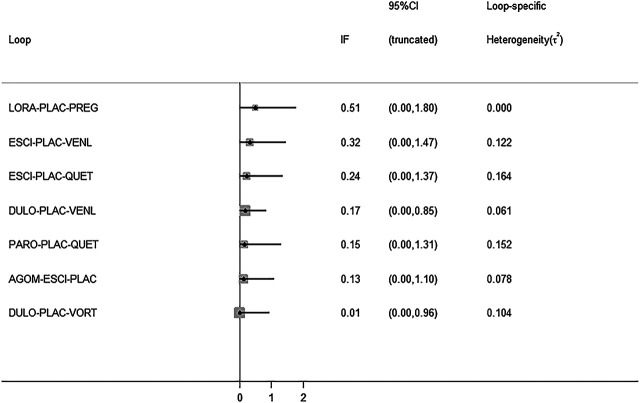
Loop inconsistency plot for remission rate.

**FIGURE 5:**
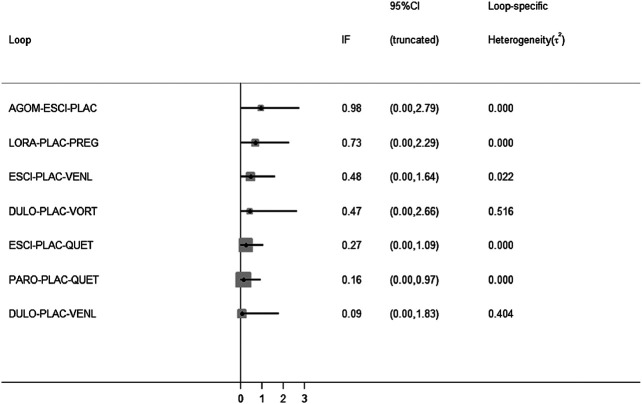
Loop inconsistency plot for tolerability.

**FIGURE 6:**
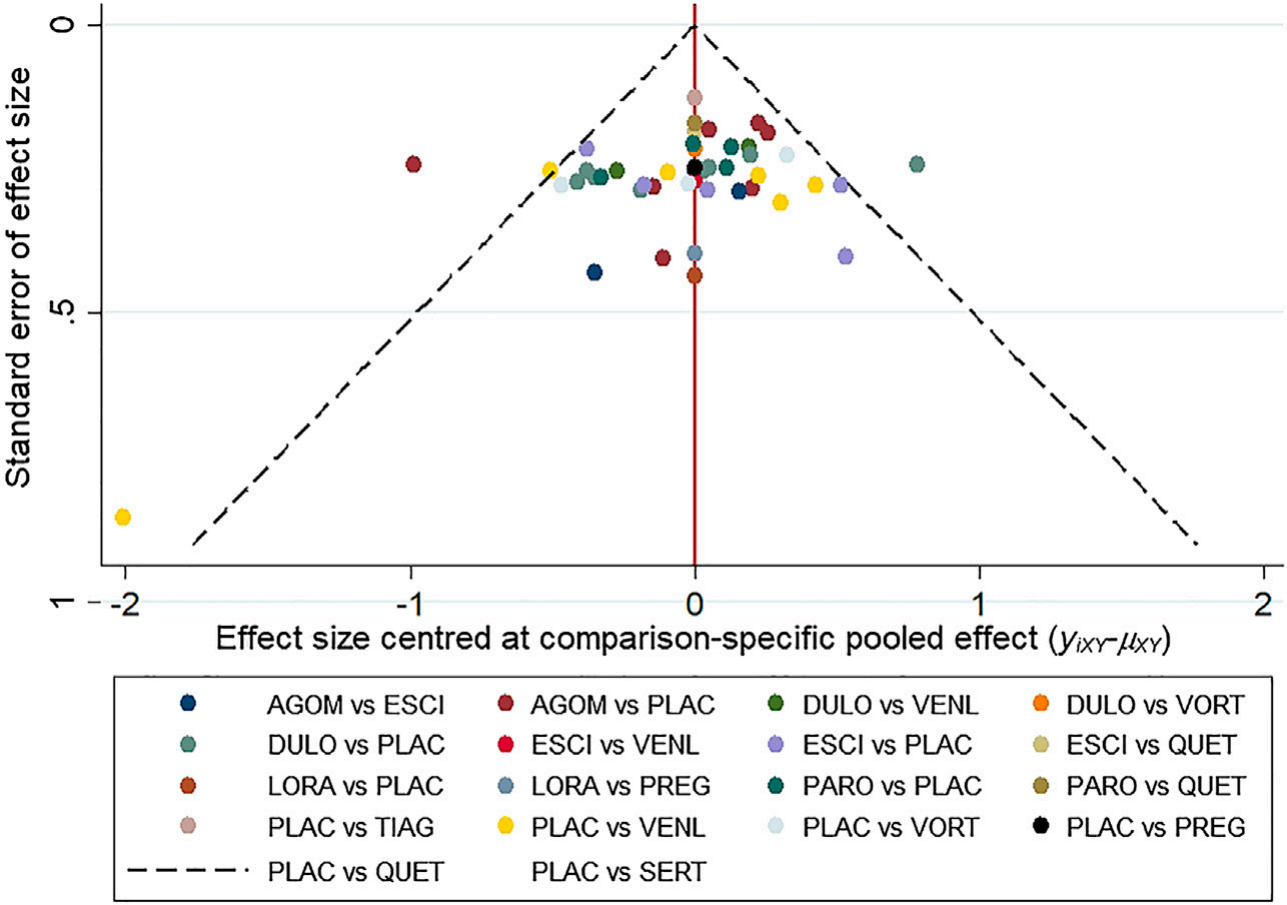
The comparison-adjusted plot for remission rate.

**FIGURE 7:**
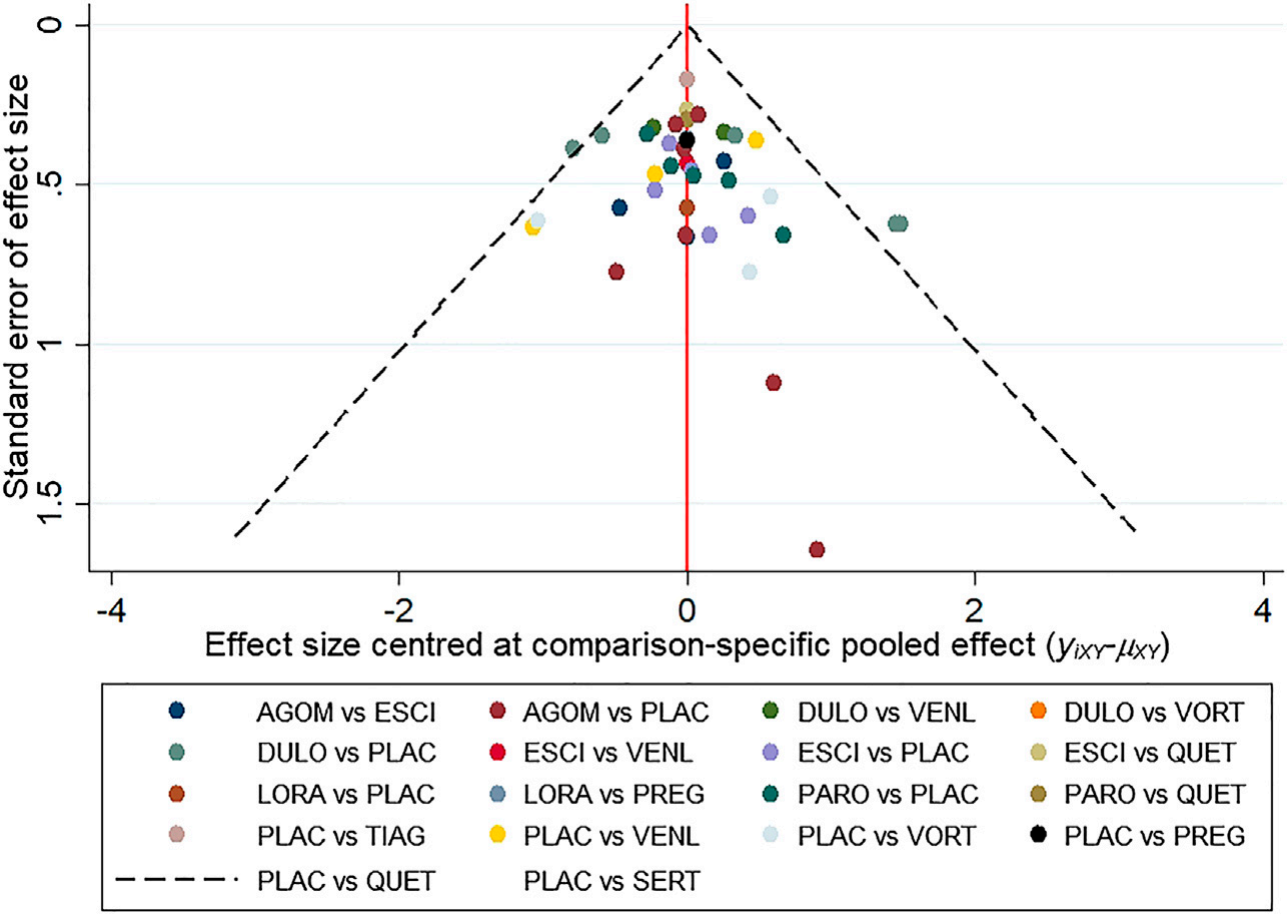
The comparison-adjusted plot for tolerability.

All active drugs were involved in at least one placebo-controlled trial. All active drugs except sertraline and tiagabine were directly compared with at least one other drug in two network plots. Three trials investigating the efficacy and safety of agomelatine were deemed to entail a low-risk quality. Trial network plots are shown in Figures 2, 3. The results of pairwise meta-analyses were generally consistent with those from network meta-analyses with regard to remission rates and tolerability. The comparative results are shown in Table 4. Both networks generated seven closed loops, and there was no inconsistency in any loop. Loop inconsistency plots are shown in Figures 4, 5. No global inconsistency was detected the within any network (*p* = 0.82 for remission rate, *p* = 0.77 for tolerability). Comparison-adjusted plots were approximately symmetric, suggesting a lack of small-study effects (Figures 6, 7).

**TABLE 4:** Comparative results for remission rate and tolerability.

Remission rate				
	Network meta-analysis	Pairwise meta-analysis		
	OR with 95% CI	Study	OR with 95% CI	Heterogeneity (%)
DULO vs. PLAC	1.88 (1.47, 2.40)	8	1.84 (1.38, 2.44)	61
PARO vs. PLAC	1.74 (1.25, 2.42)	4	1.70 (1.36, 2.13)	0
LORA vs. PLAC	1.51 (0.62, 3.70)	1	1.81 (0.77, 4.24)	—
PREG vs. PLAC	1.64 (0.89, 3.02)	2	1.59 (0.99, 2.55)	0
QUET vs. PLAC	1.88 (1.39, 2.55)	4	1.85 (1.13, 3.04)	85
VORT vs. PLAC	1.30 (0.88,1.92)	3	1.26 (0.80, 1.99)	59
AGOM vs. PLAC	2.70 (1.74, 4.19)	3	2.71 (1.91, 3.85)	0
TIAG vs. PLAC	1.13 (0.76, 1.68)	3	1.12 (0.86, 1.45)	0
PREG vs. LORA	1.09 (0.46, 2.59)	1	1.25 (0.58, 2.72)	—
ESCI vs. PLAC	2.03 (1.48, 2.78)	5	1.89 (1.31, 2.73)	54
SERT vs. PLAC	**2.01(0.99, 4.10)** ^†^	1	**2.01(1.24, 3.28)** ^†^	—
PARO vs. QUET	0.92 (0.61, 1.39)	1	1.06 (0.76, 1.48)	—
ESCI vs. QUET	1.08 (0.72, 1.61)	1	0.94 (0.65, 1.34)	—
VENL vs. PLAC	2.28 (1.69, 3.07)	6	2.42 (1.60, 3.66)	64
ESCI vs. AGOM	0.75 (0.46, 1.23)	1	1.25 (0.76, 2.06)	—
ESCI vs. VENL	0.89 (0.59, 1.34)	1	1.02 (0.60, 1.74)	—
DULO vs. VENL	0.83 (0.58, 1.17)	1	1.11 (0.73, 1.68)	—
DULO vs. VORT	1.45 (0.94, 2.23)	1	1.50 (0.99, 2.29)	—
Tolerability				
DULO vs. PLAC	2.15 (1.49, 3.11)	6	2.86 (1.34, 6.11)	76
PARO vs. PLAC	2.32 (1.56, 3.44)	4	2.17 (1.43, 3.27)	0
LORA vs. PLAC	5.98 (2.41, 14.87)	1	8.59 (2.79, 26.50)	—
PREG vs. PLAC	1.52 (0.75, 3.07)	2	1.51 (0.76, 3.00)	4
QUET vs. PLAC	4.05 (2.89, 5.65)	4	3.82 (2.70, 5.40)	0
VORT vs. PLAC	0.87 (0.49, 1.52)	3	1.19 (0.41, 3.48)	55
AGOM vs. PLAC	0.83 (0.28, 2.43)	3	1.14 (0.36, 3.65)	0
TIAG vs. PLAC	1.86 (1.25, 2.75)	1	1.85 (1.27, 2.70)	15
PREG vs. LORA	0.25 (0.12, 0.543)	1	0.28 (0.14, 0.56)	—
ESCI vs. PLAC	1.68 (1.11, 2.53)	5	1.75 (1.14, 2.70)	0
SERT vs. PLAC	0.77 (0.35, 1.66)	1	0.77 (0.38, 1.57)	—
PARO vs. QUET	0.57 (0.34, 0.90)	1	0.53 (0.30, 0.95)	—
ESCI vs. QUET	0.42 (0.27, 0.65)	1	0.38 (0.22, 0.64)	—
VENL vs. PLAC	2.25 (1.43, 3.55)	3	2.61 (1.12, 6.04)	59
ESCI vs. AGOM	2.04 (0.69, 5.88)	1	3.84 (1.04, 14.06)	—
ESCI vs. VENL	0.75 (0.43, 1.29)	1	0.50 (0.22, 1.17)	—
DULO vs. VENL	0.95 (0.62, 1.48)	1	1.34 (0.69, 2.59)	—
DULO vs. VORT	2.48 (1.45, 4.24)	1	2.75 (1.53, 4.94)	—

^†^Significant differences between pairwise analysis and network meta-analysis were in bold.

**FIGURE 8:**
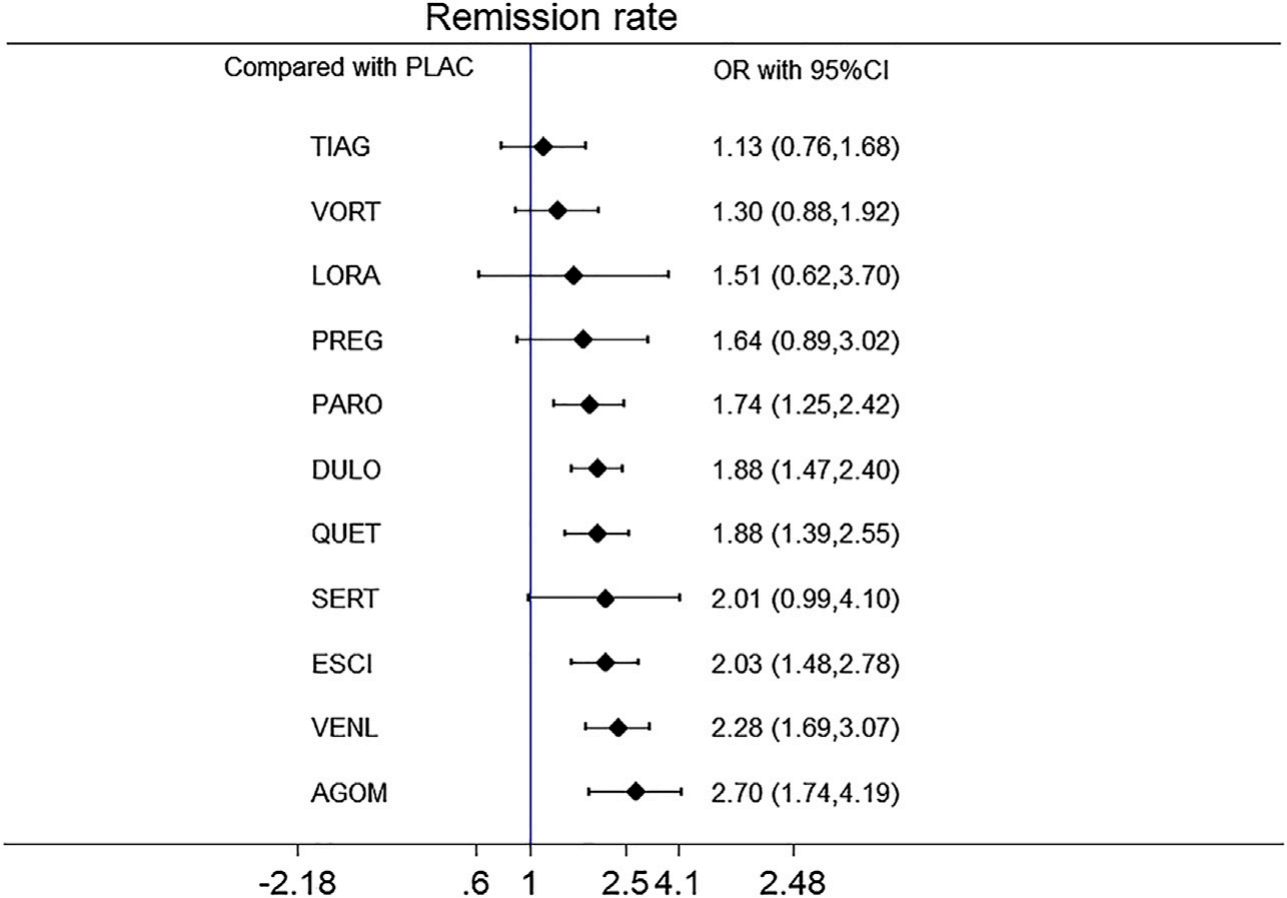
The forest plot of active drugs vs. placebo for remission rate.

**FIGURE 9:**
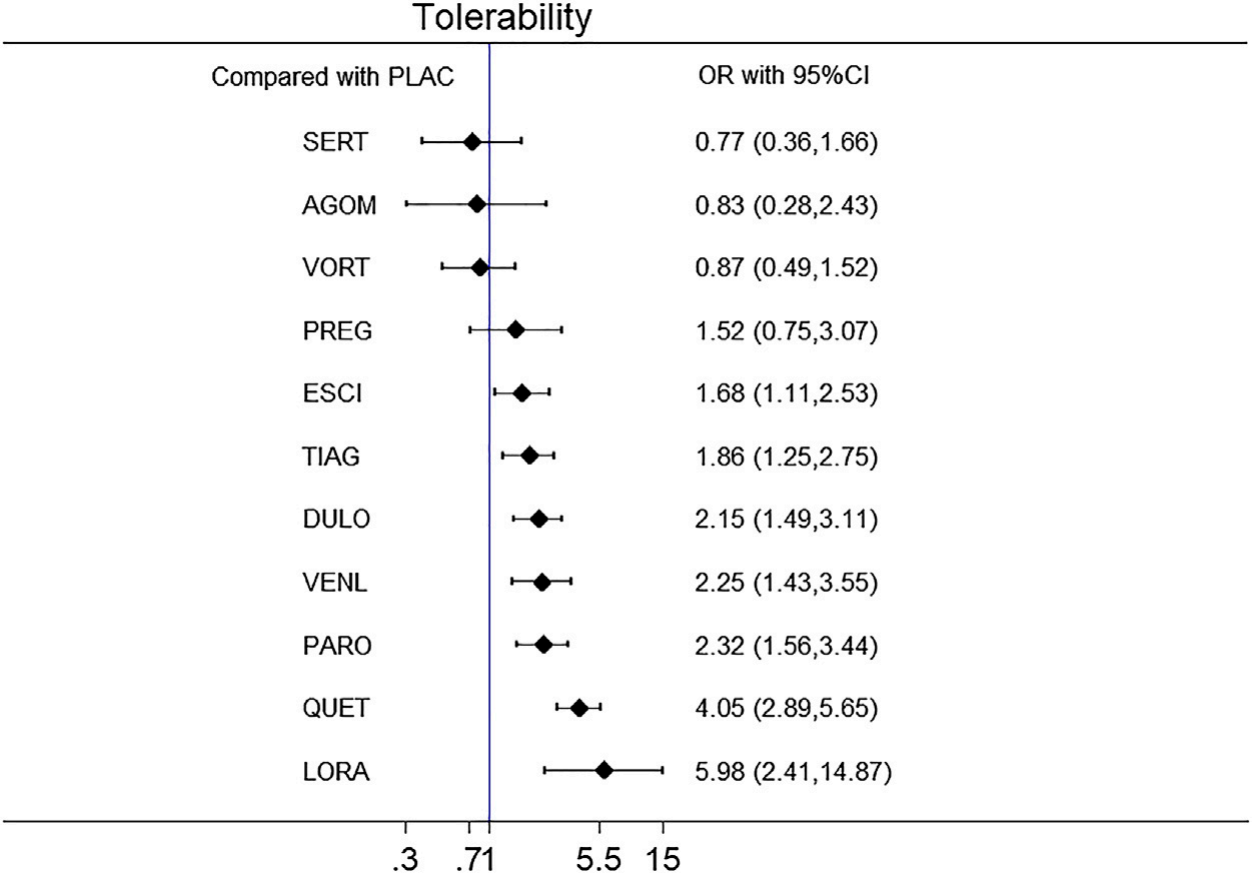
The forest plot of active drugs vs. placebo for tolerability.

A forest plot of network meta-analysis of all trials for remission rate is shown in Figure 8. With regard to remission rate (comprising 32 RCTs including 13,338 patients), agomelatine (OR 2.70, 95% CI 1.74–4.19), duloxetine (OR 1.88, 95% CI 1.47–2.40), escitalopram (OR 2.03, 95% CI 1.48–2.78), paroxetine (OR 1.74, 95% CI 1.25–2.42), quetiapine (OR 1.88, 95% CI 1.39–2.55) and venlafaxine (OR 2.28, 95% CI 1.69–3.07) were superior to placebo. Tiagabine, vortioxetine, lorazepam, and sertraline were comparable to placebo. A forest plot of network meta-analysis of all trials for tolerability is shown in Figure 9. With regard to tolerability (comprising 25 RCTs involving 12,057 patients), all of the drugs except sertraline, agomelatine, vortioxetine, and pregabalin were worse than placebo, with ORs ranging from 1.68 (95% CI 1.11–2.53) for escitalopram to 5.98 (95% CI 2.41–14.87) for lorazepam.

In head-to-head comparisons of outcomes to determine the differences between drugs, with respect to remission rate agomelatine (OR 2.08, 95% CI 1.15–3.75) and venlafaxine (OR 1.76, 95% CI 1.08–2.86) were more effective than vortioxetine. Agomelatine, duloxetine, escitalopram, quetiapine, and venlafaxine were associated with higher remission rates than tiagabine (ORs ranging between 1.66 and 2.38). With respect to tolerability quetiapine and lorazepam were worse than the other drugs, with ORs ranging between 1.75 and 7.79. The results of head-to-head comparisons for remission rate and tolerability are summarized in Table 5. The results of Bayesian analysis of remission rates were consistent with those obtained using the frequentist method, with exception of quetiapine vs. tiagabine. The results of Bayesian analysis of tolerability for agomelatine vs. venlafaxine, and tiagabine vs. sertraline or vortioxetine differed significantly from those obtained using the frequentist method. The results of sensitivity analysis are presented in Tables 6, 7. Treatments were ranked in terms of remission rate and tolerability. Agomelatine was ranked the best for remission rate, and tiagabine was ranked the worst. Sertraline was ranked the best for tolerability, and quetiapine and lorazepam were ranked the worst for tolerability. The ranking of drugs based on SUCRAs and mean ranks are shown in Table 8.

**TABLE 5:** Network meta-analysis results of head-to-head comparisons for remission rate (lower triangle) and tolerability (upper triangle).

AGOM	0.39 (0.13, 1.19)	0.49 (0.17, 1.44)	0.14 (0.03, 0.56)	0.36 (0.12, 1.11)	0.55 (0.15, 1.97)	0.20 (0.07, 0.62)	1.08 (0.29, 4.02)	0.45 (0.14, 1.39)	0.37 (0.12, 1.16)	0.96 (0.29, 3.18)
1.44 (0.87, 2.37)	**DULO**	1.28 (0.76, 2.16)	**0.36** ( **0.14, 0.95** )	0.93 (0.55, 1.57)	1.42 (0.64, 3.13)	**0.53** ( **0.33, 0.87** )	**2.79** ( **1.19, 6.53** )	1.16 (0.68, 1.96)	0.95 (0.62, 1.48)	**2.48** ( **1.45, 4.24** )
1.33 (0.81, 2.17)	0.92 (0.62, 1.37)	**ESCI**	**0.28** ( **0.10, 0.76** )	0.72 (0.42, 1.24)	1.11 (0.49, 2.49)	**0.42** ( **0.27, 0.65** )	2.18 (0.91, 5.20)	0.90 (0.52, 1.58)	0.75 (0.43, 1.29)	1.94 (0.99, 3.81)
1.78 (0.66, 4.84)	1.24 (0.49, 3.15)	1.34 (0.52, 3.47)	**LORA**	2.58 (0.96, 6.93)	**3.94** ( **1.89, 8.23** )	**0.42** ( **0.27, 0.65** )	**7.79** ( **2.32, 26.18** )	**3.22** ( **1.20, 8.64** )	2.66 (0.97, 7.30)	**6.91** ( **2.39, 19.98** )
1.55 (0.90, 2.69)	1.08 (0.72, 1.63)	1.17 (0.74, 1.84)	0.87 (0.33, 2.26)	**PARO**	1.53 (0.68, 3.42)	**0.57** ( **0.34, 0.90** )	**3.01** ( **1.27, 7.12** )	1.25 (0.72, 2.16)	2.66 (0.97, 7.30)	**2.68** ( **1.36, 5.26** )
1.64 (0.77, 3.47)	1.14 (0.59, 2.20)	1.24 (0.62, 2.45)	0.92 (0.39, 2.19)	1.06 (0.53, 2.11)	**PREG**	**0.37** ( **0.17, 0.82** )	1.97 (0.69, 5.58)	0.82 (0.37, 1.83)	0.67 (0.29, 1.55)	1.75 (0.72, 4.29)
1.43 (0.85, 2.42)	1.00 (0.68, 1.47)	1.08 (0.72, 1.61)	0.80 (0.31, 2.06)	0.92 (0.61, 1.39)	0.87 (0.44, 1.72)	**QUET**	**5.25** ( **2.27, 12.1** )	**2.18** ( **1.30, 3.65** )	**1.80** ( **1.04, 3.11** )	**4.67** ( **2.44, 8.93** )
1.34 (0.58, 3.09)	0.93 (0.44, 1.98)	1.01 (0.46, 2.20)	0.75 (0.24, 2.36)	0.86 (0.39, 1.89)	0.82 (0.32, 2.08)	0.94 (0.43, 2.03)	**SERT**	**0.42** ( **0.18, 0.98** )	**0.34** ( **0.14, 0.84** )	0.89 (0.34, 2.30)
**2.38** ( **1.32, 4.31** )	**1.66** ( **1.04, 2.65** )	**1.80** ( **1.08, 2.98** )	1.34 (0.50, 3.56)	1.54 (0.91, 2.58)	1.45 (0.70, 3.01)	**1.66** ( **1.01, 2.74** )	1.78 (0.79, 4.02)	**TIAG**	0.82 (0.46, 1.48)	**2.14** ( **1.09, 4.20** )
1.18 (0.70, 2.01)	0.83 (0.58, 1.17)	0.89 (0.59, 1.34)	0.66 (0.26, 1.71)	0.76 (0.49, 1.19)	0.72 (0.37, 1.42)	0.83 (0.54, 1.26)	0.88 (0.41, 1.91)	**0.50** ( **0.30, 0.82** )	**VENL**	**2.60** ( **1.36, 5.26** )
**2.08** ( **1.15, 3.75** )	1.45 (0.94, 2.23)	1.57 (0.95, 2.59)	1.17 (0.44, 3.10)	1.34 (0.80, 2.24)	1.27 (0.62, 2.62)	1.45 (0.89, 2.38)	1.55 (0.69, 3.50)	0.87 (0.50, 1.53)	**1.76** ( **1.08, 2.86** )	**VORT**

Drugs are reported in alphabetical order. Data are ORs (95% CI) in the column-defining treatment compared with the row-defining treatment. For remission rate, ORs higher than 1 favor the column-defining treatment. For tolerability, ORs lower than 1 favor the first drug in alphabetical order. Significant results are in bold and underscored.

**TABLE 6:** Sensitivity results for remission rate. Remission rate (Results from Bayesian method were presented in upper triangle, Results from frequentist method were presented in lower triangle).

AGOM	1.40 (0.83, 2.50)	1.30 (0.77, 2.30)	1.80 (0.63, 5.00)	1.60 (0.85, 2.90)	1.70 (0.75, 3.60)	1.40 (0.83, 2.50)	1.40 (0.56, 3.50)	2.40 (1.30, 4.70)	1.20 (0.67, 2.00)	2.10 (1.10, 3.90)	2.70 (1.70, 4.40)
1.44 (0.87, 2.37)	**DULO**	0.93 (0.58, 1.40)	1.20 (0.48, 3.10)	1.10 (0.67, 1.70)	1.20 (0.57, 2.30)	1.00 (0.64, 1.50)	0.95 (0.42, 2.20)	1.70 (1.00, 2.80)	0.81 (0.56, 1.20)	1.50 (0.90, 2.30)	1.90 (1.40, 2.50)
1.33 (0.81, 2.17)	0.92 (0.62, 1.37)	**ESCI**	1.30 (0.52, 3.50)	1.20 (0.70, 2.00)	1.20 (0.59, 2.60)	1.10 (0.70, 1.60)	1.00 (0.43, 2.40)	1.80 (1.00, 3.20)	0.86 (0.57, 1.40)	1.60 (0.90, 2.70)	2.00 (1.50, 2.90)
1.78 (0.66, 4.84)	1.24 (0.49, 3.15)	1.34 (0.52, 3.47)	**LORA**	0.88 (0.33, 2.40)	0.90 (0.35, 2.20)	0.78 (0.28, 2.00)	0.74 (0.21, 2.40)	1.30 (0.46, 3.60)	0.64 (0.23, 1.60)	1.10 (0.40, 3.10)	1.51 (0.63, 3.70)
1.55 (0.90, 2.69)	1.08 (0.72, 1.63)	1.17 (0.74, 1.84)	0.87 (0.33, 2.26)	**PARO**	1.10 (0.49, 2.20)	0.93 (0.58, 1.40)	0.87 (0.36, 2.00)	1.60 (0.87, 2.70)	0.75 (0.45, 1.20)	1.30 (0.76, 2.30)	1.80 (1.20, 2.50)
1.64 (0.77, 3.47)	1.14 (0.59, 2.20)	1.24 (0.62, 2.45)	0.92 (0.39, 2.19)	1.06 (0.53, 2.11)	**PREG**	0.86 (0.43, 1.80)	0.81 (0.30, 2.30)	1.50 (0.68, 3.10)	0.70 (0.34, 1.50)	1.30 (0.58, 2.80)	1.60 (0.87, 3.20)
1.43 (0.85, 2.42)	1.00 (0.68, 1.47)	1.08 (0.72, 1.61)	0.80 (0.31, 2.06)	0.92 (0.61, 1.39)	0.87 (0.44, 1.72)	**QUET**	0.95 (0.41, 2.20)	**1.70** ( **0.97, 2.90** )	0.81 (0.51, 1.30)	1.50 (0.84, 2.50)	1.90 (1.40, 2.70)
1.34 (0.58, 3.09)	0.93 (0.44, 1.98)	1.01 (0.46, 2.20)	0.75 (0.24, 2.36)	0.86 (0.39, 1.89)	0.82 (0.32, 2.08)	0.94 (0.43, 2.03)	**SERT**	1.80 (0.60, 5.00)	0.86 (0.37, 2.00)	1.50 (0.63, 3.80)	2.00 (0.91, 4.40)
2.38 (1.32, 4.31)	1.66 (1.04, 2.65)	1.80 (1.08, 2.98)	1.34 (0.50, 3.56)	1.54 (0.91, 2.58)	1.45 (0.70, 3.01)	**1.66** ( **1.01, 2.74** )	1.78 (0.79, 4.02)	**TIAG**	0.48 (0.28, 0.82)	0.86 (0.46, 1.60)	1.10 (0.73, 1.80)
1.18 (0.70, 2.01)	0.83 (0.58, 1.17)	0.89 (0.59, 1.34)	0.66 (0.26, 1.71)	0.76 (0.49, 1.19)	0.72 (0.37, 1.42)	0.83 (0.54, 1.26)	0.88 (0.41, 1.91)	0.50 (0.30, 0.82)	**VENL**	1.80 (1.00, 3.10)	2.40 (1.70, 3.30)
2.08 (1.15, 3.75)	1.45 (0.94, 2.23)	1.57 (0.95, 2.59)	1.17 (0.44, 3.10)	1.34 (0.80, 2.24)	1.27 (0.62, 2.62)	1.45 (0.89, 2.38)	1.55 (0.69, 3.50)	0.87 (0.50, 1.53)	1.76 (1.08, 2.86)	**VORT**	1.30 (0.87, 2.00)
2.70 (1.74, 4.19)	1.88 (1.47, 2.40)	2.03 (1.48, 2.78)	1.51 (0.62, 3.70)	1.74 (1.25, 2.42)	1.64 (0.89, 3.02)	1.88 (1.39, 2.55)	2.01 (0.99, 4.10)	1.13 (0.76, 1.68)	2.28 (1.69, 3.07)	1.30 (0.88.1.92)	**PLAC**

Results from Bayesian method were presented in upper triangle, Results using frequentist method were presented in lower triangle.Data are ORs (95% CI) in the column-defining treatment compared with the row-defining treatment. For remission rate, ORs higher than 1 favour the column-defining treatment. For tolerability, ORs lower than 1 favour the first drug in alphabetical order. Significant results are in bold and underscored.

**TABLE 7:** Sensitivity results for tolerability. Tolerability (Results from Bayesian method were presented in upper triangle, Results using frequentist method were presented in lower triangle).

AGOM	0.32 (0.12, 1.00)	0.43 (0.17, 1.40)	0.13 (0.03, 0.59)	0.34 (0.12, 1.20)	0.52 (0.16, 1.70)	0.20 (0.07, 0.60)	1.10 (0.27, 4.60)	0.42 (0.14, 1.30)	0.31 (0.11, 0.96)	0.78 (0.25, 2.70)	0.82 (0.31, 2.30)
0.39 (0.13, 1.19)	**DULO**	1.40 (0.81, 2.30)	0.38 (0.14, 1.00)	1.00 (0.57, 1.80)	1.50 (0.64, 3.40)	0.59 (0.35, 0.99)	3.10 (1.30, 8.30)	1.30 (0.71, 2.30)	0.94 (0.61, 1.50)	2.50 (1.20, 4.30)	2.40 (1.70, 3.50)
0.49 (0.17, 1.44)	1.28 (0.76, 2.16)	**ESCI**	0.28 (0.09, 0.77)	0.74 (0.43, 1.30)	1.10 (0.46, 2.50)	0.42 (0.25, 0.71)	2.40 (0.87, 6.20)	0.90 (0.49, 1.70)	0.70 (0.39, 1.20)	1.80 (0.86, 3.50)	1.70 (1.20, 2.70)
0.14 (0.03, 0.56)	0.36 (0.14, 0.95)	0.28 (0.10, 0.76)	**LORA**	2.90 (0.98, 7.80)	4.00 (1.60, 9.50)	1.60 (0.54, 4.50)	8.80 (2.60, 33.00)	3.50 (1.20, 9.60)	2.50 (0.90, 7.20)	6.30 (2.20, 18.00)	6.30 (2.40, 17.00)
0.36 (0.12, 1.11)	0.93 (0.55, 1.57)	0.72 (0.42, 1.24)	2.58 (0.96, 6.93)	**PARO**	1.40 (0.60, 3.40)	0.56 (0.35, 0.97)	3.30 (1.20, 8.20)	1.20 (0.67, 2.30)	0.90 (0.50, 1.90)	2.40 (1.20, 4.60)	2.40 (1.50, 3.70)
0.55 (0.15, 1.97)	1.42 (0.64, 3.13)	1.11 (0.49, 2.49)	3.94 (1.89, 8.23)	1.53 (0.68, 3.42)	**PREG**	0.38 (0.17, 0.93)	2.20 (0.67, 6.70)	0.88 (0.35, 2.00)	0.62 (0.27, 1.50)	1.60 (0.64, 4.10)	1.60 (0.77, 3.60)
0.20 (0.07, 0.62)	0.53 (0.33, 0.87)	0.42 (0.27, 0.65)	0.42 (0.27, 0.65)	0.57 (0.34, 0.90)	0.37 (0.17, 0.82)	**QUET**	5.60 (2.10, 14.00)	2.20 (1.20, 3.90)	1.60 (0.92, 3.00)	4.20 (2.00, 8.00)	4.20 (2.80, 6.10)
1.08 (0.29, 4.02)	2.79 (1.19, 6.53)	2.18 (0.91, 5.20)	7.79 (2.32, 26.18)	3.01 (1.27, 7.12)	1.97 (0.69, 5.58)	5.25 (2.27, 12.1)	**SERT**	**0.39** ( **0.15, 1.10** )	0.31 (0.11, 0.78)	0.78 (0.26, 2.10)	0.74 (0.32, 1.80)
0.45 (0.14, 1.39)	1.16 (0.68, 1.96)	0.90 (0.52, 1.58)	3.22 (1.20, 8.64)	1.25 (0.72, 2.16)	0.82 (0.37, 1.83)	2.18 (1.30, 3.65)	**0.42** ( **0.18, 0.98** )	**TIAG**	0.77 (0.38, 1.50)	**2.00** ( **0.94, 3.80** )	1.90 (1.20, 3.00)
**0.37** ( **0.12, 1.16** )	0.95 (0.62, 1.48)	0.75 (0.43, 1.29)	2.66 (0.97, 7.30)	2.66 (0.97, 7.30)	0.67 (0.29, 1.55)	1.80 (1.04, 3.11)	0.34 (0.14, 0.84)	0.82 (0.46, 1.48)	**VENL**	2.60 (1.10, 5.20)	2.50 (1.50, 4.10)
0.96 (0.29, 3.18)	2.48 (1.45, 4.24)	1.94 (0.99, 3.81)	6.91 (2.39, 19.98)	2.68 (1.36, 5.26)	1.75 (0.72, 4.29)	4.67 (2.44, 8.93)	0.89 (0.34, 2.30)	**2.14** ( **1.09, 4.20** )	2.60 (1.36, 5.26)	**VORT**	0.96 (0.60, 1.90)
0.83 (0.28, 2.43)	2.15 (1.49, 3.11)	1.68 (1.11, 2.53)	5.98 (2.41, 14.87)	2.32 (1.56, 3.44)	1.52 (0.75, 3.07)	4.05 (2.89, 5.65)	0.77 (0.35, 1.66)	1.86 (1.25, 2.75)	2.25 (1.43, 3.55)	0.87 (0.49, 1.52)	**PLAC**

Results from Bayesian method were presented in upper triangle, Results using frequentist method were presented in lower triangle.Data are ORs (95% CI) in the column-defining treatment compared with the row-defining treatment. For remission rate, ORs higher than 1 favour the column-defining treatment. For tolerability, ORs lower than 1 favour the first drug in alphabetical order. Significant results are in bold and underscored.

**TABLE 8:** The ranking of all treatments.

Outcomes	Treatments	SUCRA	Mean rank
Remission rate	Agomelatine	89.7	2.1
	Venlafaxine	77.2	3.5
	Escitalopram	67.1	4.6
	Sertraline	64	5.0
	Duloxetine	57.6	5.7
	Quetiapine	58.6	5.9
	Paroxetine	49.2	6.6
	Pregabalin	46.3	6.9
	Lorazepam	41.2	7.5
	Vortioxetine	23.9	9.4
	Tiagabine	19	9.9
	Placebo	6.2	11.3
Tolerability	Sertraline	88.2	2.3
	Vortioxetine	85.6	2.6
	Agomelatine	82.9	2.9
	Placebo	79.8	3.2
	Pregabalin	57.2	5.7
	Escitalopram	52.3	6.2
	Tiagabine	46.0	6.9
	Duloxetine	35.2	8.1
	Paroxetine	30.7	8.6
	Venlafaxine	31.6	8.6
	Quetiapine	7.4	11.2
	Lorazepam	3.2	11.7

## Discussion

To our knowledge the current analysis constitutes the most up-to-date evidence with respect to comparisons of remission rate associated with pharmacological treatments obtained by pooling direct and indirect comparisons. A similar network meta-analysis was published by Baldwin D. S. et al., 2011 (Baldwin D. et al., 2011) but in the current study the newest interventions including agomelatine and vortioxetine were analyzed after a more broad-reaching search. Thus, the results of the present analysis may inform clinicians about how to choose appropriate treatments when various therapies are available.

Strict eligibility criteria ensured that high-quality studies were included in the meta-analysis. Trials that included patients with comorbidities were excluded to ensure the similarity assumption of network meta-analysis. Furthermore, the exclusion of studies of GAD with comorbidities allows us to speculate that the anxiolytic effect of drugs in GAD is independent from their effects on comorbidities. Consistency with reference to the similarity of different sources of evidence is an important component when evaluating the reliability and accuracy of network meta-analyses (Higgins et al., 2012; He et al., 2019) and in the present analysis no inconsistency between the overall results and the results of pairwise analysis was evident. These advantages strengthen the reliability and validity of our conclusions.

Paroxetine, duloxetine, quetiapine, escitalopram, venlafaxine, and agomelatine were better than placebo as determined by HAM-A scores ≤7. Patients administered these treatments may ultimately experience minimal symptoms of anxiety and achieve a full recovery after completing follow-up duration. Notably, however, of these six drugs only agomelatine was well tolerated. Other drugs did not exhibit superiority over a placebo. Paroxetine, duloxetine, quetiapine, escitalopram, venlafaxine, and lorazepam exhibited poor tolerability as defined by withdrawal due to adverse events. Tiagabine was the poorest with regard to remission rates, and agomelatine and venlafaxine were more efficacious than vortioxetine.

Currently most guidelines and meta-analyses recommend SSRIs and SNRIs as the first-line pharmacotherapies for GAD. In current analysis venlafaxine (six trials including 2,218 patients), duloxetine (8 trials including 3,392 patients), escitalopram (five trials including 2,093 patients) and paroxetine (four trials including 1,594 patients) were good in terms of remission and were comparable to other drugs in terms of tolerability, which is consistent with previous meta-analyses (Baldwin D. et al., 2011; He et al., 2019). Sertraline, recommended as first choice for GAD by the National Institute for Health and Care Excellence, was not significantly superior to placebo in terms of remission on the basis of the only relevant study in the present analysis (Allgulander et al., 2004). In contrast, in pairwise analysis sertraline was favorable in terms of remission rate. A small sample population may have reduced the accuracy of estimates for sertraline. It is hoped further trials will clarify conclusions pertaining to sertraline in the future. It was ranked the best in terms of tolerability in the current network meta-analysis, which is concordant with Baldwin D. S. et al., 2011 (Baldwin D. et al., 2011).

Comorbidity is common in GAD in clinical practice with approximately 62.4% of patients suffering from comorbid major depression and approximately 39.5% exhibiting dysthymia (Judd et al., 1998). Therefore, GAD subjects with the two major comorbidities can be treated with SSRIs or SNRIS. Although SSRIs and SNRIs are widely considered the first choice for GAD, their slow onset of action and unfavorable side effects including increased risk of bleeding of the gastrointestinal tract preclude their application in some patients (Tyrer et al., 2006; Carvalho et al., 2016; Laporte et al., 2017).

In the current analysis quetiapine (four trials including 3,036 patients) yielded better remission than placebo but exhibited worse tolerability than placebo or other drugs, which is consistent with previous meta-analyses (Stein et al., 2011; Maneeton et al., 2016). Quetiapine may be considered as an alternative treatment in patients with GAD comorbid with sleep disturbance because it can reportedly reduce the symptoms of anxiety and improve sleep (Monti and Monti, 2000; Maneeton et al., 2016). Agomelatine is currently approved for the treatment of GAD and major depressive disorder. It has a unique mechanism of action and functions as a melatonin receptor agonist on MT_1_ and MT_2_, and as a selective serotonin receptor antagonist on 5-HT_2C_ receptors, which confers its capacity to treat relevant disorders (Millan et al., 2003; Guardiola-Lemaitre et al., 2014). Among the drugs included in the current analysis, agomelatine (three trials including 938 patients) had the largest effect on remission and exhibited the relatively good tolerability. Given its benefits, agomelatine may be an attractive option for the treatment of GAD with concurrent depression and insomnia. Unfortunately, in a systematic review published by Freiesleben et al. agomelatine associated with a markedly higher rate of liver injury than placebo, paroxetine, sertraline, escitalopram, and fluoxetine (Freiesleben and Furczyk, 2015). Hepatotoxicity may limit its use in practice.

Benzodiazepines are frequently used to treat GAD. Only one relevant RCT (including 191 patients) was included in the present analysis, and in that trial lorazepam was not superior to placebo or other drugs with regard to remission, and it exhibited the poorest tolerability. Disadvantages including a lack of the capacity to alleviate depression, dependence and side effects, together with poor tolerability, impede the use of benzodiazepines to treat anxiety disorders. In practice clinicians tend to combine an antidepressant and a benzodiazepine, then the benzodiazepine is gradually tapered off when the antidepressant shows effectiveness (Tyrer et al., 2006; Baldwin D. S. et al., 2011).

In the current analysis tiagabine (three trials including 1,791 patients) and pregabalin (two trials including 464 patients) yielded remission rate that were slightly but not statistically significantly better than placebo. Tiagabine was the poorest in terms of remission, which is consistent with the work of Baldwin D. S. et al., 2011 (Baldwin D. et al., 2011). Vortioxetine, a multimodal antidepressant, has been licensed for major depressive disorder since 2013. In a systematic review and meta-analysis of four placebo-controlled trials including GAD patients vortioxetine exhibited no superiority over placebo in terms of remission, and it was well tolerated (Qin et al., 2019), which is consistent with the results of the present analysis. Agomelatine and venlafaxine were better than vortioxetine in the current analysis.

There are, however, some important limitations. The substantial heterogeneity between the trials hindered some comparisons. Differences stemming from both baseline demographic characteristics and trial designs contribute to this existence of heterogeneity, particularly with regard to comorbidities and the severity of GAD. The analysis did not exclude patients with low-to-moderate depression or other anxiety disorders comorbid with GAD given that these comorbidities are common in clinical practice. Notably, however, it is extremely hard to determine the extent to which these comorbidities affected the results. The meta-analysis intentionally did not include unpublished studies, and this may have resulted in a degree of associated bias. Most of the studies included were sponsored by pharmaceutical manufactures, and this may have resulted in some reporting bias. Some studies with small samples reduced the strength and validity of some treatment comparisons. For example, only one eligible study was identified for sertraline and lorazepam, two studies for pregabalin, and three studies for agomelatine, vortioxetine and tiagabine. Studies with small samples may lead to conflicting results with regard to some comparisons for tolerability due to comparatively sensitivity. Thus, conclusions pertaining to these drugs should be drawn and interpreted conservatively. The vast majority of patients included in the current network meta-analysis were Caucasian, thus it is uncertain whether the findings are applicable to other ethnic groups. The use of the last observation carried forward approach and high dropout rates in some studies potentially resulted in attrition bias. Only short-term treatment was included (median duration 8 weeks), whereas GAD is known to be a chronic disorder that requires long-term treatment. Noteworthily, in a previous study has demonstrated that early improvement of GAD was associated with endpoint remission (Pollack et al., 2008a; Pollack et al., 2008b). We suspect that achieving remission via acute treatment is more beneficial to patients with GAD in long-term treatment.

In summary, the findings of this network meta-analysis constitute the latest evidence to consider when contemplating viable treatment options for GAD with respect to remission and tolerability profiles. All comparisons between current drugs should be considered within the context of the limitations of this network meta-analysis and patient’s specific situations. We hope that this meta-analysis provides helpful perspectives facilitating informed decisions by patients and clinicians.

## Author Contributions

WK and HD designed this study. WK and XW selected the studies, extracted data and analyzed data. WK, JW, YZ, and YLZ drafted this manuscript. BS checked this manuscript.

## Conflict of Interest

The authors declare that the research was conducted in the absence of any commercial or financial relationships that could be construed as a potential conflict of interest.

## Abbreviations

**Abbreviations:** PLAC, Placebo; DULO, Duloxetine; PARO, Paroxetine; SERT, Sertraline; ESCI, Escitalopram; VENL, Venlafaxine; QUET, Quetiapine; VORT, Vortioxetine; AGOM, Agomelatine; TIAG, Tiagabine; LORA, Lorazepam; PREG, Pregabalin.
